# Chain Graph Models to Elicit the Structure of a Bayesian Network

**DOI:** 10.1155/2014/749150

**Published:** 2014-02-05

**Authors:** Federico M. Stefanini

**Affiliations:** Dipartimento di Statistica, Informatica, Applicazioni “G. Parenti”, Università degli Studi di Firenze, Viale Morgagni 59, 50134 Firenze, Italy

## Abstract

Bayesian networks are possibly the most successful graphical models to build decision support systems. Building the structure of large networks is still a challenging task, but Bayesian methods are particularly suited to exploit experts' degree of belief in a quantitative way while learning the network structure from data. In this paper details are provided about how to build a prior distribution on the space of network structures by eliciting a chain graph model on structural reference features. Several structural features expected to be often useful during the elicitation are described. The statistical background needed to effectively use this approach is summarized, and some potential pitfalls are illustrated. Finally, a few seminal contributions from the literature are reformulated in terms of structural features.

## 1. Introduction

Bayesian networks (BNs) are possibly the most successful graphical models to represent probabilistic and causal relationships [1, 2]. BNs are used in very different fields including medical domains [3], engineering [4], ecology [5], bioinformatics [6], and many others.

The core of this class of models is made by a directed acyclic graph (DAG) *𝒢*, where nodes in the graph are labels of modeled variables (elements of vector **X**), and oriented edges (arrows) capture probabilistic and/or causal relationships. The joint distribution of **X** is represented by the product of conditional distribution functions following from the structure of *𝒢*. If substantial prior information is available on a given problem domain, it is possible that an expert defines the structure of *𝒢* and even the parameters inside the conditional distribution functions at a reasonable extent. Otherwise, structure and parameters have to be estimated from data (structural and parameter learning), for example, from a collection of exchangeable observations *𝒟* = (**x**_1_,…, **x**_*n*_).

It is often the case that an expert knows some features of DAG *𝒢*, but knowledge is not enough to define a DAG because several of its aspects are affected by relevant uncertainty. Following the Bayesian paradigm [7, 8], the expert is invited to state his/her degree of belief about *𝒢* by eliciting a prior distribution on the set of DAGs which can be considered given a fixed set of variables (nodes). All other unknown quantities, like missing values and parameters, are considered in the prior distribution [9].

Learning the structure of a BN remains nowadays still challenging for the combinatorial explosion of candidate structures with the increase in the number of considered nodes. Following Robinson [10], the number of possible DAGs on 6 nodes is 3781503; thus the enumeration of all structures while eliciting expert beliefs is unfeasible. Computational difficulties in the full enumeration of DAGs follow from about a dozen of nodes on. For these reasons, several restrictions and simplifications in stating prior beliefs were considered in the past, with the aim of making structural learning tasks affordable in large networks. Widely adopted elicitation techniques are based on restrictions like a total ordering of nodes, the presence of sharp order constraints on nodes, the marginal independence of unknowns, or the existence of a prior network which is a good summary of expert beliefs.

In a recent work [11], graphical models were proposed to elicit beliefs about the structure of a BN. The approach is characterized by the possibility of expressing beliefs about limited (but relevant) aspects of the problem domain, called structural features [12], that by their own nature are often only indirectly related to oriented edges in the unknown DAG.

Here the most general approach based on chain graph (CG) models is reconsidered with the aim of establishing connections with seminal contributions from the literature on structural learning, of detailing a general parameterization, and of describing one way to perform the refinement of elicited beliefs. Common useful structural features are defined and some issues related to their implementation and revision are examined.

## 2. Methods

Notation and some background information on graphs and Markov properties are provided below. A comprehensive account may be found in [13–15]. An approach to the elicitation of structural features by CG models is described, together with methods for the revision.

### 2.1. Graphs

A graph *𝒢* is a pair (*V*, *E*) where *V* = {*v*_1_, *v*_2_,…, *v*_*K*_} is a finite set of nodes and *E* ⊂ *V* × *V* is the set of edges. The set *E* represents the structure of the graph because it defines which nodes are linked by an edge and if such edge is directed (arrow) or not. If (*v*_*i*_, *v*_*j*_) ∈ *E* and (*v*_*j*_, *v*_*i*_) ∈ *E* then an undirected edge joins *v*_*i*_ and *v*_*j*_, indicated as *v*_*i*_—*v*_*j*_, and *v*_*j*_ is neighbor of *v*_*i*_. If (*v*_*i*_, *v*_*j*_) ∈ *E* but (*v*_*j*_, *v*_*i*_) ∉ *E* the ordered pair corresponds to the directed edge *v*_*i*_ → *v*_*j*_; *v*_*j*_ is said to be the child of *v*_*i*_ and *v*_*i*_ is a parent of *v*_*j*_. The set pa(*v*_*j*_) includes all parents of node *v*_*i*_, that is, all nodes originating arrows with end in *v*_*i*_, while the set ch(*v*_*i*_) collects all children of *v*_*i*_, namely, all nodes in which arrows originated from *v*_*i*_ end.

A path is a sequence of vertices such that there is an edge for each pair of subsequent nodes in the sequence, that is, *v*_*i*_—*v*_*i*+1_ or *v*_*i*_ ← *v*_*i*+1_ or *v*_*i*_ → *v*_*i*+1_. A directed path is a path in which all edges maintain the head-to-tail orientation, for example, (*v*_*i*_, *v*_*j*_, *v*_*k*_) with *v*_*i*_ → *v*_*j*_ → *v*_*k*_.

In a directed graph all edges are directed. The ancestors an(*v*_*i*_) of node *v*_*i*_ are nodes on a directed path reaching *v*_*i*_, while descent nodes de(*v*_*i*_) are nodes on a directed path starting from node *v*_*i*_. Note that *v*_*i*_ ∈ de(*v*_*i*_) and that *v*_*i*_ ∈ an(*v*_*i*_). The extension of the above definition to an(*A*) with *A* ⊂ *V* is obtained by union of sets an(*v*_*i*_) for each *v*_*i*_ ∈ *A*. A similar extension holds for de(*A*).

In a directed graph without cycles it is not possible to visit the same node more than one time by following a directed path, and in this case the graph is called directed acyclic graph. A moralized DAG is an undirected graph obtained by joining pairs of nodes sharing children (if not yet connected) with an undirected edge and by removing the direction of all edges. A subgraph *𝒢*_*A*_ on *A* ⊂ *V* is obtained by removing all nodes in *V*∖*A* and all edges in which at least one node is in *V*∖*A* from the graph *𝒢*.

A graph without directed edges is called undirected graph (UG). An UG with *E* = *V* × *V* is said to be complete. A subgraph on a subset *S* ⊂ *V* of nodes is obtained by removing nodes not in *S* and all edges reaching-leaving nodes not in *S*. Note that a subset of nodes is indicated by capital letters. A clique *C* is a maximal complete subgraph of an UG.

A chain graph, also called partially directed acyclic graph (PDAG), is made by an ordered collection of chain components *τ* = (*τ*_1_, *τ*_2_,…) which are undirected graphs and by directed edges between nodes located in different chain components, so that the arrow *v*_*i*_ → *v*_*j*_ is allowed only if *v*_*i*_ belongs to a chain component preceding the chain component in which *v*_*j*_ is located. Therefore directed edges are forbidden within a chain component and in the direction from *τ*_*j*_ to *τ*_*j*−*k*_, with *k* > 0.

The moralization of a chain graph mimics the moralization of a DAG. A moralized CG, indicated as *𝒢* ^*m*^, is obtained by the following steps:

1. let *k* = 2;
2. join with undirected edges all pairs of nodes in pa(*τ*_*k*_), with pa(*τ*_*k*_) being the union of parents sets for each node in chain component *τ*_*k*_;
3. iterate step (2) for *k* = 3,4,…;
4. remove directions to all edges.

### 2.2. Some Markov Properties

Conditional independence [16] is fundamental to reason out highly structured stochastic systems and to simplify the representation of high dimensional distributions.

In this paper the random vector **X** refers to random variables included in the BN. The notation used hereafter is based on nodes in *V*; thus **X** is also indicated as *X*_*V*_ = *X*_*v*_1_,…,*v*_*K*__ = (*X*_*v*_1__,…, *X*_*v*_*K*__) and the sample space *Ω*_*X*_*V*__ = ⊗_*v*_*i*_∈*V*_ *Ω*_*X*_*v*_*i*___ is the Cartesian product of sample spaces of considered variables.

The joint probability distribution of a random vector *X*_*V*_ is Markov with respect to an UG *𝒢* if

(1) $$\begin{array}{c} p\left(x_{v_{1}},x_{v_{2}},\ldots ,x_{v_{K}}\right)=\sigma^{-1}\prod_{C\in 𝒞}\phi_{C}\left(x_{C}\right) \end{array}$$

with *𝒞* being a collection of graph cliques in *𝒢* and with *ϕ*_*C*_ nonnegative functions called clique potentials; note that *x*_*C*_ is the coordinate projection of vector *x*_*V*_ on the subset of coordinates defined by *C*; *σ* = ∑_*Ω*_*X*_*V*___∏_*C*∈*𝒞*_ *ϕ*_*C*_(*x*_*C*_) is the partition function that normalizes the product of potentials in (1).

Markov properties for positive distributions with respect to an undirected graph may be read using the separation theorem [14]. Let *A* ⊂ *V*, *B* ⊂ *V*, *S* ⊂ *V* be disjoint subsets of nodes. The separation theorem states that subvectors *X*_*A*_ and *X*_*B*_ are conditionally independent given the subvector *X*_*S*_ if and only if all paths from a node in *A* to a node in *B* include nodes located in *S*; thus nodes in *S* separate nodes in *A* from nodes in *B*.

The joint probability distribution of random variables indexed in *V* is Markov with respect to a DAG *𝒢* if the following factorization holds:

(2) $$\begin{array}{c} p\left(x_{v_{1}},x_{v_{2}},\ldots ,x_{v_{K}}\right)=\prod_{v_{i}\in V}p\left(x_{v_{i}}\;|\;x_{\text{pa}(v_{i})}\right), \end{array}$$

where *x*_pa(*v*_*i*_)_ is the random vector made by variables whose labels belong to the parents set of *v*_*i*_.

Markov properties on a DAG may be read using the separation theorem for directed graphs [14]. Given three disjoint sets of nodes *A*, *B*, *S*, consider the subgraph *𝒢* defined on an(*A* ∪ *B* ∪ *S*) after moralization, say *𝒢*_an(*A*,*B*,*S*)_^*m*^. Random subvectors *X*_*A*_ and *X*_*B*_ are conditionally independent given the subvector *X*_*S*_ if and only if nodes in *S* separate nodes in *A* from nodes in *B* in *𝒢*_an(*A*,*B*,*S*)_^*m*^.

A joint probability distribution is Markov with respect to a CG *𝒢* if

(3) $$\begin{array}{c} p\left(x_{v_{1}},x_{v_{2}},\ldots ,x_{v_{K}}\right)=\prod_{\tau_{i}\in \tau }p\left(x_{\tau_{i}}\;|\;x_{\text{pa}(\tau_{i})}\right) \end{array}$$

with *τ*_*i*_ ∈ *τ*, the chain components of *𝒢*. Furthermore factors on r.h.s. of (3) may be factorized by considering the subgraph on nodes defined by *τ*_*i*_ ∪ pa(*τ*_*i*_):

(4) $$\begin{array}{c} p\left(x_{\tau_{i}}\;|\;x_{\text{pa}(\tau_{i})}\right)=\sigma_{t}^{-1}\prod_{C\in 𝒞}\phi_{C}\left(x_{C}\right), \end{array}$$

where *σ*_*t*_^−1^, *t* ∈ *Ω*_pa(*τ*_*i*_)_ are normalization constants, one for each conditioning value of the random subvector *X*_pa(*τ*_*i*_)_. The working UG in (4) is obtained by removing the orientation of edges from parents of nodes in *τ*_*i*_ and by joining them into a complete undirected subgraph.

Conditional independence relationships in CGs are also obtained from an extension of the separation theorem in UGs and DAGs for positive distributions [17]. Let *𝒢* be a chain graph and *A* ⊂ *V*, *B* ⊂ *V*, *S* ⊂ *V* be three disjoint subsets of *V*. The separation theorem states that subvectors *X*_*A*_ and *X*_*B*_ are conditionally independent given the subvector *X*_*S*_ if and only if all paths from a node in *A* to a node in *B* in *𝒢*_an(*A*∪*B*∪*S*)_^*m*^ include nodes of *S*; thus nodes in *S* separate nodes in *A* from nodes in *B*. Note that *𝒢*_an(*A*∪*B*∪*S*)_^*m*^ is the moral graph of the smallest ancestral set for *A* ∪ *B* ∪ *S*, that is, a subgraph of *𝒢* ^*m*^ described in Section 2.1.

### 2.3. Causal DAGs

A DAG may represent causal relations among variables. According to the causal semantic, an arrow *v*_*i*_ → *v*_*j*_ indicates that *v*_*i*_ is a direct cause of *v*_*j*_ with respect to nodes included in *V*, that is, at the considered model granularity. In principle the intervention on variable *x*_*v*_*i*__ may bear an effect on *x*_*v*_*j*__. The intervention on a subset of variables *D* ⊂ *V* indicates the external setting of variables in *X*_*D*_ to prescribed values; thus the system or process is perturbed, not merely observed.

Pearl [2, pp. 27–32] starts with the definition of functional causal models, which are deterministic in nature, and he demonstrates [2, theorem 1.4.1] that such formulation induces the Markov factorization in (2), the so-called Markov causal assumption. An equivalent representation embeds exogenous variables into the node of interest and transforms the deterministic relationships into probabilistic conditioning, thus leading to Bayesian networks.

A key property of a casual DAG *𝒢* is the stability under external intervention: if a variable *x*_*v*_*i*__ is manipulated all the other variables maintain their relationships as represented by *𝒢*. In other terms the intervention is local on manipulated variables and it does not break all the other relationships represented in a causal DAG. The intervention regime is in contrast with the plain observation of values taken by the random vector *X*_*V*_.

The granularity of a causal DAG depends on the variables included in the model. A variable *X*_*L*_ not included in a causal DAG may eventually affect just one variable *X*_*v*_, with *v* ∈ *V*; otherwise if several variables are affected then a more general class of models is needed, called semi-Markovian networks (not considered further in this work).

For an updated presentation of the approach see [18] while [19] warns against the blind definition of DAGs within the causal semantic in observational studies. He also reconsiders the foundations of causal inference by anchoring them to the extended conditional independence (C.I.), both at algebraic level and with a graphical counterpart based on influence diagrams.

### 2.4. Inference about the Structure of Bayesian Networks

In all cases in which strong prior information is absent, structural learning is performed by means of a database *𝒟* = (*x*_1_,…, *x*_*n*_) of *n* exchangeable observations of the random vector *X*_*V*_.

Several algorithms have been proposed to infer causal and probabilistic relations, but in Bayesian inference key quantities enter into the joint probability distribution of *𝒟* and network's unknowns given the context *ξ*:

(5) $$\begin{array}{c} p\left(𝒟,\theta ,z\;|\;\xi \right)=p\left(𝒟\;|\;\theta ,z,\xi \right)\cdot p\left(\theta \;|\;z,\xi \right)\cdot p\left(z\;|\;\xi \right), \end{array}$$

where *θ* = (*θ*_*v*_1_,pa(*v*_1_)_,…, *θ*_*v*_*K*_,pa(*v*_*K*_)_) are vectors of parameters characterizing the conditional probability distributions of *X*_*v*_*i*__ given *X*_pa(*v*_*i*_)_; variable *Z* indicates the unknown DAG, and it is built as a bijection from the set of DAGs (fixed *V*) to a subset *Ω*_*Z*_ of natural numbers.

The likelihood function *p*(*𝒟* | *θ*, *z*, *ξ*) is typically expressed as a product of multinomials by using sufficient statistics. Whenever expert beliefs are reasonably captured by Dirichlet prior distributions for elements of *θ*, and they are elicited as marginally independent, closed-form integration marginalizes out thetas. The resulting marginal distribution *p*(*𝒟* | *z*, *ξ*) has a reduced dimensionality and may be optimized with respect to *z* while looking for optimal structures characterized by the highest posterior probability values [9]:

(6) p(𝒟,z ∣ ξ)=∫p(𝒟 ∣ θ,z,ξ)·p(θ ∣ z,ξ)·p(z ∣ ξ)·dθ=p(z ∣ ξ)·∏i=1K∏j=1qiΓ(αi,j)Γ(αi,j+Ni,j)   ·∏s=1riΓ(αi,j,s+Ni,j,s)Γ(αi,j,s)

with *r*_*i*_ being the number of states taken by *X*_*i*_ and *q*_*i*_ of *X*_pa(*v*_*i*_)_; sufficient statistics are *N*_*i*,*j*,*s*_ for the *i*th variable taking the *s*th state while its parent configuration is in the *j*th state; *α*_*i*,*j*_ = ∑_*s*=1_^*r*_*i*_^ *α*_*i*,*j*,*s*_ and *N*_*i*,*j*_ = ∑_*s*=1_^*r*_*i*_^ *N*_*i*,*j*,*s*_. A clever choice of hyperparameters guarantees the likelihood equivalence; that is, all BNs equivalent as regards the set of encoded conditional independence relationships are equally scored by (6).

Equation (6) shows that the elicitation of the prior distribution *p*(*z* | *ξ*) is a key step to perform Bayesian structural learning using *𝒟*.

### 2.5. Plausible Network Features

A candidate structure *z* ∈ *Ω*_*Z*_ on a fixed set of nodes *V* is plausible if it has got structural features (SFs) believed to be relevant by the expert. Following Stefanini [11, Definition  1], we recall the formal definition.

**Definition 1 (SF)**

A structural feature (SF) *ℛ*_*j*_(*z*, *w*) in a reference set *ℛ* for the set of DAGs on *V* is a predicate describing a plausible probabilistic or causal characteristic of the unknown directed acyclic graph *z* ∈ *Ω*_*Z*_. Argument *w* is in the partition *𝒲* of a given numeric domain *Ω*_*W*_ of variable *W*. An atomic structural feature (ASF) *ℛ*_*j*_(*z*) does not depend on any auxiliary variable *W*.

A reference set *ℛ* is a collection *ℛ* = {*ℛ*_*j*_ : *j* ∈ *J*} of SFs indexed in a set *J*, with *n*_*f*_ being the number of considered SFs. A proposition might be defined to carry disbelief to a candidate structure, but the feature-rises-belief direction is conveniently adopted here.

An example makes the previous definition operational. Let us define *ℛ*_1_(*z*, *w*) = “The number of immediate causes of *X*_*v*_3__ is in *w*” to capture the expert belief about the number of parents of node *v*_3_ in the unknown DAG. An expert may consider *Ω*_*W*_ = {0,1,…, 12} and *𝒲* = ({0}, {1,2, 3}, {4,5}, {6,…, 12}). Given a candidate structure *z*, its plausibility will be determined by the element *w* ∈ *𝒲* that makes the predicate true. A simple representation of configurations taken by reference features is obtained by descriptors [11, Definition  2].

**Definition 2 (descriptors)**

A descriptor *R*_*i*_ for the SF *ℛ*_*i*_ is a map:

(7) $$\begin{array}{c} R_{i}:{𝒲}_{i}\times \left\{\text{false},\text{true}\right\}⟶\left\{0,1,2,\ldots ,\left|{𝒲}_{i}\right|\right\} \end{array}$$

so that (*w*, false) ↦ 0 for all *w* ∈ *𝒲*_*i*_ and (*w*, true) ↦ *h*_*w*_ for all *w* ∈ *𝒲*_*i*_; that is, a different integer is associated with each *w* if true. The vector *R* = (*R*_1_, *R*_2_,…, *R*_*n*_*f*__) defined on the Cartesian product *Ω*_*R*_ = ⊗_*i*=1_^*n*_*f*_^ *Ω*_*R*_*i*__ = ⊗_*i*=1_^*n*_*f*_^{0,1,…, |*𝒲*_*i*_|} is called vector of descriptors. The descriptor of an ASF is defined by false ↦ 0 and true ↦ 1.

The *j*th configuration of descriptors in *R* is indicated as *r*_*j*_ = (*r*_1,*j*_, *r*_2,*j*_,…, *r*_*n*_*f*_,*j*_) ∈ *Ω*_*R*_ while a generic configuration is indicated as *r* ∈ *Ω*_*R*_. The *j*th configuration of a subvector of *R* defined by indexes in *A* is *r*_*A*,*j*_; for example, *r*_{1,3},*j*_ = (*r*_1,*j*_, *r*_3,*j*_).

Vector *R* induces equivalence classes on *Ω*_*Z*_; that is, *𝒵* = {*𝒵*_*r*_ : *r* ∈ *Ω*_*R*_} contains sets of structures, with *𝒵*_*r*_ made by all those DAGs sharing the same configuration *r*.

Note that members of the same equivalence class must be associated with the same degree of belief by the principle of insufficient reason: they differ in irrelevant and unconsidered ways by construction.

Despite the generality of Definition 1 some features are expected to occur more often than others. Below, some of them are described without pretending to be exhaustive.

#### 2.5.1. Indegree and Outdegree

In applications characterized by a small sample size, it is useful to impose a sharp constraint during the greedy search of top-scored candidate structures. The maximum number of arrows entering into a node, the indegree, is set to a small integer, say 2 to 5, to exclude structures with a large number of parents from the consideration. A large number of parents implies a CPT with a huge number of parameters which are affected by large uncertainty after conditioning to observed data because of sampling zeros. A similar constraint may be set on the number of arrows leaving a node.

Two reference features naturally embed this kind of information.

- “The maximum number of arrows reaching a node is in *n*_*id*_ for all nodes in *V*,” where *n*_*id*_ is a small set of integers close to 1 elicited from the expert.
- “The maximum number of arrows leaving a node is in *n*_*od*_ for all nodes in *V*,” where *n*_*od*_ is a small set of integers close to 1 elicited from the expert.

The above two features may be exploited to increase the plausibility of candidate structures which are sparsely connected. Higher control on connectivity is obtained by considering the fraction of nodes showing a given degree of connectivity, as described below.

#### 2.5.2. Partitioned Connectivity

A different way of characterizing the connectivity is obtained by eliciting the minimum fraction of nodes in DAG showing a given number of parents (children) and by iterating the elicitation from 0 parents (children) up to a small integer *s*.

Let *s* be a small integer representing the maximum number of parents (children) to be considered. Let *W* = (*W*_0_,…, *W*_*s*_) be a vector of nondecreasing numbers in [0,1], with

(8) ΩW={(ω0,ω1,ω2,…,ωb,…,ωs):0≤ωb≤ωb+1<ωs=1}

being the sample space. The elicitation of this feature is based on a vector *a* = (*a*_0_, *a*_1_, *a*_2_,…, *a*_*b*_,…, *a*_*s*_), with 0 ≤ *a*_*b*_ ≤ *a*_*b*+1_ < *a*_*s*_ = 1, and on the induced partition *𝒲* = {*w*_0_, *w*_1_} where

(9) $$\begin{array}{c} w_{1}=\left\{\left(\omega_{0},\omega_{1},\ldots ,\omega_{s}\right):\omega_{b}\geq a_{b},b=0,1,\ldots ,s\right\} \end{array}$$

and *w*_0_ = *Ω*_*W*_∖*w*_1_.

Two reference features naturally embed the extended evaluation of connectivity:

- “The *a*-partitioned inconnectivity of degree *s* is in *w*_1_,” with *a*, *s*, and *w*_1_ defined above; a candidate structure *z* shows this feature if the cumulative relative frequency *F*_*b*_ of nodes in *z* with number of parents equal or less than *b* is greater than *a*_*b*_; that is, *F*_*b*_ ≥ *a*_*b*_, *b* = 1,2,…, *s*.
- “The *a*-partitioned outconnectivity of degree *s* is in *w*_1_,” with *a*, *s*, and *w*_1_ defined above; a candidate structure *z* shows this feature if the cumulative relative frequency *F*_*b*_ of nodes in *z* with number of children equal or less than *b* is greater than *a*_*b*_; that is, *F*_*b*_ ≥ *a*_*b*_, *b* = 1,2,…, *s*.

Trained experts could prefer a conventional total number of nodes equal to 100 to elicit cumulative percentages instead of cumulative fractions of nodes.

In Table 1, an example is shown where *s* = 5 and the elicited vector *a* is defined on fractions. A candidate structure has the partitioned inconnectivity feature if the fraction of root nodes is equal or above 0.01, while the proportion of nodes with at least 1 parent is equal or above 0.2 and so on.

#### 2.5.3. Direct Cause and Direct Effect

The reference feature “The variable *x*_*v*_*i*__ is an immediate cause of variable *x*_*v*_*j*__” refers to *x*_*v*_*i*__ as a parent of *x*_*v*_*j*__ so that by setting (intervening on) the value of the variable *x*_*v*_*i*__ to a given value, the distribution of *X*_*v*_*j*__ is modified. This relation holds at the level of selected granularity; thus it may change if the collection of variables (nodes in *V*) is modified.

#### 2.5.4. Causal Ancestors

The “direct cause” feature may be extended by considering a variable *x*_*v*_*i*__ which is on a causal (directed) path reaching node *x*_*v*_*j*__. In this case the reference feature is “The variable *x*_*v*_*i*__ is an indirect cause of variable *x*_*v*_*j*__”; thus the expert believes that one or more variables mediate the effect of *x*_*v*_*i*__ on *x*_*v*_*j*__.

#### 2.5.5. Causal Hubs

A hub node in a network is characterized by a high number of arrows leaving it. A reference feature which captures the local connectivity of node *v*_*i*_ is “Node *v*_*i*_ is a hub node of at least outdegree *w*,” with the outdegree indicating the number of arrows originated in *v*_*i*_ and *w* a set of integers. Note that the defined feature is a localized version of the outdegree feature.

The expert might believe that a hub node should be present, but without indicating a specific node. In this case the *a*-partitioned outconnectivity feature (with a large *s*) can be exploited for this purpose.

#### 2.5.6. Conditional Independence Relationships

A statement about C.I. among three disjoint subsets of random variables may take the following form: “The random vector *X*_*A*_ is conditionally independent from the random vector *X*_*B*_ given vector *X*_*S*_,” with *A*, *B*, *S* being disjoint subsets of nodes in *V*.

### 2.6. The Degree of Belief

The prior distribution on the space of structures on *V* is obtained by “extending the argument;” that is,

(10) $$\begin{array}{c} P\left[Z=z\;|\;\xi \right]=\sum_{r\in \Omega_{R}}P\left[Z=z\;|\;R=r,\xi \right]\cdot P\left[R=r\;|\;\xi \right]. \end{array}$$

By recognizing that *R* induces the partition *𝒵*, it follows that

(11) $$\begin{array}{c} p\left(z\;|\;\xi \right)=\frac{1}{n_{r^{\left[z\right]}}}\cdot P\left[R=r^{\left[z\right]}\;|\;\xi \right]=\frac{p\left(r_{1}^{\left[z\right]},r_{2}^{\left[z\right]},\ldots ,r_{n_{f}}^{\left[z\right]}\;|\;\xi \right)}{n_{r^{\left[z\right]}}}, \end{array}$$

where *n*_*r*^[*z*]^_ is the cardinality of the equivalence class in which *z* is located and where the structural configuration of DAG *z* is *r* ^[*z*]^. In [12] the size of each equivalence class *𝒵*_*r*_ was estimated by Monte Carlo simulation to face the combinatorial explosion in the number of DAGs to be enumerated with the increase of the number of nodes:

(12) $$\begin{array}{c} \left|{\hat{𝒵}}_{r}\right|=\frac{N_{V}\left(N_{r}+1\right)}{N_{T}+1}, \end{array}$$

where *N*_*T*_ is the total number of DAGs uniformly sampled from the space of all DAGs on *V*, *N*_*V*_ is the size of such space (see [10]), and *N*_*r*_ ≤ *N*_*T*_ is number of sampled DAGs showing configuration *r*.

The numerator on the right of (11) represents the joint belief on each configuration of descriptors. While the elicitation in full generality becomes cognitively and numerically overwhelming around 7 descriptors on, some parsimony is achieved if a small number of descriptors may be considered at one time, so that conditional independence relationships among descriptors may be exploited. This is a choice available to the expert through the definition of an order relation on descriptors.

**Definition 3 (ordered partition)**

The ordered partition

(13) $$\begin{array}{c} 𝒪=\left({𝒪}_{1},{𝒪}_{2},\ldots \right) \end{array}$$

of descriptors is defined by the expert to indicate disjoints subsets of SFs to be jointly considered during the elicitation, from the first subset *𝒪*_1_ to the last.

Otherwise stated, the expert decomposes the whole elicitation problem following an order taken from the substantive content of the specific problem domain: features are grouped according to the priority in the elicitation.

The elicitation of the ordered partition is performed by formulating questions in the language typical of a given problem domain. It is indeed difficult to define those questions in general terms, because they result to be quite abstract and they are likely to be obscure for the domain expert (see [11]). We assume here that questions are properly phrased and that an ordered partition is defined.

If a strict order relation is elicited, each element of *𝒪* contains one descriptor: this is a special case addressed in [20]. Another special case is represented by a trivial partition of just one subset that contains all descriptors [21]. In the two special cases above it is possible to define, respectively, a Bayesian network and a Markov network on descriptors. The general case made by several subsets, each one of cardinality two or more, was addressed by using CG models in [11] for ASFs. Below details are provided about a reference set of nonatomic features.

The joint probability distribution of descriptors *p*(*r*_1_, *r*_2_,…, *r*_*n*_*f*__ | *𝒪*, *ξ*) is assumed to be Markov with respect to the elicited partition where descriptors within group *𝒪*_*j*_ define the chain component *τ*_*j*_ of the CG model (see (3)). The elicitation keeps on by asking in the language of the domain expert which descriptors in subset *𝒪*_*j*_ should be jointly considered while defining the degree of belief. Nodes corresponding to related descriptors are joined by undirected edges and the collection of cliques defining the chain component are found. The elicitation is iterated for all elements in the ordered partition *𝒪*.

The resulting CG may support the elicitation if model parameters are cognitively suited for the quantitative step, that is, if they are interpretable and easy to assess for the expert, at least after some training.

The first chain component is elicited as a marginal distribution which is not conditioned on other descriptors. An undirected graphical model on descriptors in *𝒪*_1_ is defined through the multiplicative model in (4), under empty conditioning. Nevertheless some care is needed because potential functions on cliques are not uniquely defined. Here a log-linear parameterization is suggested, following [13]. For example, if the first chain component has just one clique made by three descriptors, say *R*_1_, *R*_2_, *R*_3_, we define the multiplicative model as below, after exponentiation and rearrangement of log-linear terms:

(14) p(r1,r2,r3 ∣ ξ)p(0,0,0 ∣ ξ)  =ϕ1(r1)ϕ2(r2)ϕ3(r3)ϕ1,2(r1r2)   ×ϕ1,3(r1r3)ϕ2,3(r2r3)ϕ1,2,3(r1r2r3).

Thus the odds value with respect to the no-feature configuration *r*_1,2,3_ = (0,0, 0) is explained by a multiplicative model where each factor, for example, *ϕ*_2,3_(*r*_2_ *r*_3_), is equal to one if one or more descriptors are null, otherwise they are positive (the so-called treatment parameterization).

The elicitation in (14) is performed on the odds scale by asking the domain expert how many times the configuration (*r*_1_, *r*_2_, *r*_3_) is more plausible than (0,0, 0) for descriptors *R*_1_, *R*_2_, *R*_3_. This question is iterated for all configurations in the Cartesian product *Ω*_*R*_1__ × *Ω*_*R*_2__ × *Ω*_*R*_3__ after exclusion of (0,0, 0). Questions are posed from single main effects towards higher order interactions terms, so that one factor at a time is considered (see algorithm below).

The procedure is iterated for all cliques in the first CG component *τ*_1_, and indeed factors already elicited in previously considered cliques are not reconsidered anymore. For example, the chain component *R*_1_—*R*_2_—*R*_3_ is made by two cliques, *R*_1_, *R*_2_ and *R*_2_, *R*_3_; thus the shared factor *ϕ*_2_(*r*_2_) is elicited just one time.

The general algorithm for the first chain component is summarized below.

(1) Consider the undirected graph on descriptors elicited as the first chain component. Control questions include the following: “Are all the relevant features included in the elicitation?”, “Are all pairs of features jointly affecting the probability of a structure linked by an undirected edge?”. If needed, revise the order relation and (or) links within chain component.
(2) Check out that each descriptor *R*_*v*_*i*__ given its neighbors *R*_ne(*v*_*i*_)_ is independent of all other descriptors in the first CG component.
(3) Find the cliques of such UG (model generator) [13].
(4) For each clique, elicit parameter values by using odds.
  (i) Elicit main effects {*ϕ*_*i*_(*r*_*i*,*j*_)}, one at a time for all configurations, by assigning an odds value with respect to the baseline with no features; that is,
    (15) $$\begin{array}{c} \phi_{i}\left(r_{i,j}\right)=\frac{p\left(0,\ldots ,0,r_{i,j},0,\ldots ,0\;|\;\xi \right)}{p\left(0,\ldots ,0\;|\;\xi \right)}. \end{array}$$
   A control question for this step is: “How much above one is the odds value for the sole presence of feature *R*_*i*_ = *r*_*i*,*j*_ > 0?”
  (ii) Elicit first order interactions {*ϕ*_*i*,*l*_(*r*_*i*,*j*′_, *r*_*l*,*j*′′_)}, one at a time, by assigning a multiplicative term for each configuration under the question: “Which is the value of the multiplicative term {*ϕ*_*i*,*l*_(*r*_*i*,*j*′_, *r*_*l*,*j*′′_)} needed to account for the interaction of feature *R*_*i*_ = *r*_*i*,*j*′_ > 0 with feature *R*_*l*_ = *r*_*l*,*j*′′_ > 0?” The expression helping in this step is
    (16) p(0,…,ri,j′,0,…,rl,j′′,0,…,0 ∣ ξ)p(0,…,0 ∣ ξ)  =ϕi(ri,j′)ϕl(rl,j′′)ϕi,l(ri,j′,rl,j′′),
   where *ϕ*_*i*_(*r*_*i*,*j*′_), *ϕ*_*l*_(*r*_*l*,*j*′′_) are already elicited; this is the cross product ratio of features *R*_*i*_, *R*_*l*_, after dividing by *ϕ*_*i*_(*r*_*i*,*j*′_), *ϕ*_*l*_(*r*_*l*,*j*′′_). It is clear that if the interaction is absent *ϕ*_*i*,*l*_(*r*_*i*,*j*′_, *r*_*l*,*j*′′_) = 1, while *ϕ*_*i*,*l*_(*r*_*i*,*j*′_, *r*_*l*,*j*′′_)∈(0,1) means that the interaction reduces the plausibility and *ϕ*_*i*,*l*_(*r*_*i*,*j*′_, *r*_*l*,*j*′′_) > 1 raises the plausibility of the considered configuration.
  (iii) Iterate the step above with higher order interaction terms (for all configurations) with two constraints: before moving to higher interaction terms all the terms of the same degree must have been already elicited; moreover the maximum degree of interaction among a subset of features is defined by the size of the clique they belong to (model generator). For example, with the interaction of order two {*ϕ*_*i*,*l*,*k*_(*r*_*i*,*j*′_, *r*_*l*,*j*′′_, *r*_*k*,*j*′′′_)}, the control question might be: “Having already elicited values of *ϕ*_*i*_(*r*_*i*,*j*′_), *ϕ*_*l*_(*r*_*l*,*j*′′_), *ϕ*_*k*_(*r*_*k*,*j*′′′_), *ϕ*_*i*,*l*_(*r*_*i*,*j*′_, *r*_*l*,*j*′′_), *ϕ*_*i*,*k*_(*r*_*i*,*j*′_, *r*_*k*,*j*′′′_), and *ϕ*_*l*,*k*_(*r*_*l*,*j*′′_, *r*_*k*,*j*′′′_), which is the value of the multiplicative term {*ϕ*_*i*,*l*,*k*_(*r*_*i*,*j*′_, *r*_*l*,*j*′′_, *r*_*k*,*j*′′′_)} needed to adjust the odds value after considering the interaction among features whose configurations are *r*_*i*,*j*′_ > 0, *r*_*l*,*j*′′_ > 0, *r*_*k*,*j*′′′_ > 0?”; the helper expression is
    (17) p(0,…,ri,j′,…,rl,j′′,…,rk,j′′′,…,0 ∣ ξ)p(0,…,0 ∣ ξ)  =Q ϕi,l,k(ri,j′,rl,j′′,rk,j′′′),
   where factor
    (18) Q=ϕi(ri,j′)ϕl(rl,j′′)ϕk(rk,j′′′)ϕi,l(ri,j′,rl,j′′)×ϕi,k(ri,j′,rk,j′′′)ϕl,k(rl,j′′,rk,j′′′)
   is already elicited. Similar expressions follow straightforwardly for higher order interactions.
(5) Calculate the baseline probability *p*(0,…, 0 | *ξ*) of a structure without features by exploiting ∑_*r*∈*Ω*_*R*__ *p*(*r* | *ξ*) = 1.
(6) Revise the elicited values following the guidelines of Section 2.7.
(7) Move to the next chain component.

The algorithm presented above may be also applied to chain components *τ*_2_, *τ*_3_,…, that is, with a nonempty set of parents. The elicitation of parameters in a conditional Markov network is performed by iterating the algorithm for the first chain component over each configuration of conditioning parents. It follows that the elicitation burden depends on the number of parents, more precisely, on the cardinality of the Cartesian product where factors are samples spaces of parents.

The algorithms defined above lead to a prior distribution on configurations, but the elicitation does not end before the revision of elicited values takes place.

### 2.7. Implementation and Revision Issues

The flexibility achieved by defining predicates entails potential pitfalls that should be considered during an elicitation run with structural features.

The revision of elicited beliefs is always needed because the expert is not expected to provide unbiased elicited values, especially with limited training or in complex problem domains. Revision and elaboration of the prior distribution [22, and references therein] are applied to control elicitation bias and other causes of poor elicitation.

Overelicitation is an important practice to check for the presence of elicited quantities that do not reflect the expert degree of belief. Nevertheless, in large networks elaboration of elicited probability values is the main resource for checking the quality of elicitation. In the proposed framework, the elicitation through reference features produces proper probability distributions; therefore one elaboration consists of inspecting one or more margins of the probability distribution *p*(*r*_1_,…, *r*_*n*_*f*__ | *ξ*), for example, the bivariate margin *p*(*r*_*i*_, *r*_*j*_ | *ξ*), to look for configurations whose plausibility causes surprise or disbelief in the expert. This operation is particularly meaningful if inspected margins do not straightforwardly relate to elicited odds, for example, taking descriptors belonging to different cliques. Surprise and disbelief ask for the revision of elicited values. If an association between selected margins and bias is suspected, random selection of margins is an option.

The stability of elicited values against different order relations on descriptors (reference features) should not be assumed. Nevertheless, in complex problem domains overelicitation made by the repetition of the interview with other ordered partitions not only seems unacceptable as regards the work load, but it could even be cognitively unfeasible, for example, if the original ordered partition selected by the expert is induced by a scientific hypothesis.

The core of the proposed approach is based on predicates representing structural features. The expert might believe that some configurations of features are plausible although they are incompatible with DAGs, for example, because they imply the presence of cycles. A positive probability value would be assigned to such a configuration r*, but this is not an instance of elicitation bias if the elicited distribution properly matches expert beliefs. If this is the case, *p*(*z* | *ξ*) = 0 because *P*[*Z* = *z* | *R* = r*] = 0.

The way a predicate is specified determines the granularity of the elicitation and the cardinality of equivalence classes in *𝒵*. For example, let us consider two nodes *v*_*i*_ ∈ *V* and *v*_*j*_ ∈ *V* and the reference feature *ℛ*_1_ = “Nodes *v*_*i*_, *v*_*j*_ are not descendent of other nodes in *V*_*R*_”.

Reworking the original proposition we have two simpler predicates:

- *ℛ*_1′_ = “Node *v*_*i*_ is not a descendent of other nodes in *V*_*R*_”;
- *ℛ*_1′′_ = “Node *v*_*j*_ is not a descendent of other nodes in *V*_*R*_.”

and they can be considered by conjunction. In Table 2, the relation between the original reference feature (right) and the conjoint components (left) is shown: ¬*ℛ*_1_ collects three configurations generated by the conjunction of simpler predicates. It follows that simpler predicates are needed if the expert degree of belief changes over collapsed configurations in *ℛ*_1_, that is, ¬*ℛ*_1′_∩*ℛ*_1′′_, *ℛ*_1′_∩¬*ℛ*_1′′_, ¬*ℛ*_1′_∩¬*ℛ*_1′′_. While nothing prevents the expert from defining rich predicates, care should be taken to select a granularity suited to properly represent expert beliefs.

There are indeed several different ways of formulating a predicate. If two reference sets of features induce the same set of equivalence classes *𝒵* then they are operationally equivalent. Nevertheless, from a cognitive standpoint, substantial differences in the ease of elicitation might depend on the way propositions are formulated [22]. Let us consider the reference feature *ℛ*_1_ = “Nodes *v*_*i*_, *v*_*j*_ precede all other nodes.” Does the expert use “precede” as “come before” all other nodes in the order relationship of nodes in *V*? Given a DAG with *v*_*j*_ disconnected from other nodes how to answer? Does the expert use “precede” meaning “are ancestors of all other nodes in *V*” or to indicate that those nodes do not receive arrows coming from other nodes? This example makes clear that some training is mandatory in order to make an expert effective in the elicitation: a trained expert is expected to choose the right granularity in the elicitation and to define meaningful predicates, that is, statements straightforwardly true/false when applied to any DAG defined on *V*.

Several reference features are jointly considered in actual applications, a number typically far beyond what the expert may simultaneously consider with success. Thus there is the possibility that exclusion and implication relations are not recognized. For example, let us consider two features: *ℛ*_3_ = “The indegree is three or less” and *ℛ*_25_ = “*v*_*i*_ is a sink node.” After reworking the last feature, the expert reformulates the statement as “Node *v*_*i*_ has indegree ten or more.” Clearly the plausibility of *ℛ*_3_∧*ℛ*_25_ should be null; otherwise the actual interpretation of *ℛ*_3_ is “The indegree of all but *v*_*i*_ is three or less.”

Implication must be recognized to maintain the probabilistic coherence; for example, let *ℛ*_3_ = “Variable *X*_*v*_*i*__ is a direct cause of *X*_*v*_*j*__” and *ℛ*_5_ = “Variable *X*_*v*_*i*__ is an ancestral cause of *X*_*v*_*j*__” be two reference features. Clearly *ℛ*_3_ implies *ℛ*_5_ and the joint plausibility of both features is bound to the plausibility of *ℛ*_3_. In a normalized reference set logical relationships among features are properly handled.

## 3. Results and Discussion

A few seminal approaches to the elicitation of beliefs on structures are reconsidered as special cases in the proposed framework based on structural features. A published case study on breast cancer (BC case-study) [23] will be (partially) exploited at illustrative purposes. The whole set of nodes *V* includes: age (AGE), the proliferative index-marker Ki67/MIB-1 (PROLN), oestrogen receptors (ER), progesterone receptors (PR), the receptor tyrosine kinase HER2/neu (NEU), and the P53 protein.

### 3.1. Buntine 1991

In the seminal paper of Buntine [24], a prior distribution on the set of DAGs for a fixed set *V* is defined by assuming a total ordering of nodes in the context *ξ*. The probability of the parent set for node *v* is defined by the product of probability for events like “There is an edge *y* → *v*,” shortly *P*["*y* → *v*"], extended to each *y* preceding *v* in the order relation. The subjective probability value elicited for a network structure *z* is calculated by marginal independence of parent sets. The original formalization defines a total ordering ≺, so that if *y*≺*x*_*i*_ it may belong to the parent's set Π_*i*_ of *x*_*i*_; then a full specification of beliefs for each edge in the directed graph is needed and measured in units of subjective probability; finally the independence of parent sets (Π_1_,…, Π_*n*_) is assumed. The distribution on the set of structures is ([24], modified)

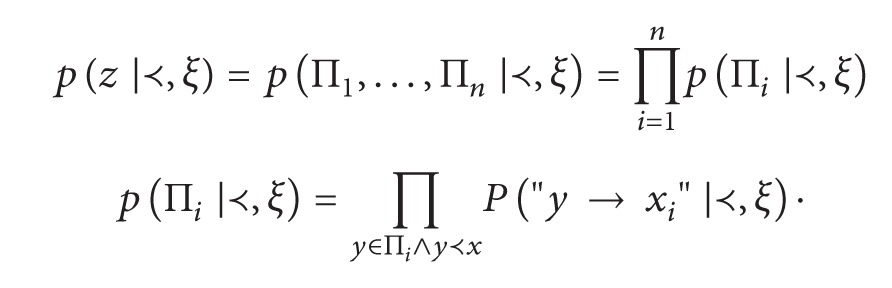
(19)

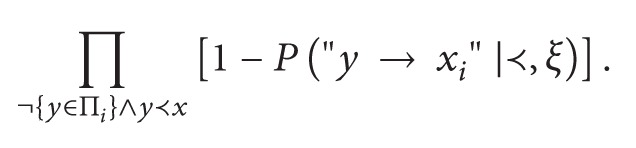
(20)
Given the order relation (*v*_3_, *v*_4_, *v*_1_, *v*_2_) on four nodes, *n* = 4, parent sets are Π_3_ = *∅*, Π_4_⊆{*y*_*v*_3__}, Π_1_⊆{*y*_*v*_3__, *y*_*v*_4__}, Π_2_⊆{*y*_*v*_3__, *y*_*v*_4__, *y*_*v*_1__}.

Despite the huge importance of Buntine's seminal work [24], some limitations should be underlined. Under the causal semantic of BNs, the expert might fail in stating such node order which defines the “causal flow” along nodes. In large regulatory networks of system biology a lot of assignments are expected to be 0.5 because expert beliefs may involve a small subset of arrows. We also expect that some plausibility assignments depend on what is already assigned, for example, due to biological substantive laws, but the need of such conditioning is not accounted for. Finally, we remark that several node orders are compatible with the same sparse DAG on *V*; therefore the specification of a strict order should not be enforced.

The above example may be straightforwardly cast in terms of reference features. The order relation is part of the context *ξ*, and we define the set of reference features *R*_*i*,*j*_ = “An arrow is from *v*_*i*_ to *v*_*j*_,” with all possible pairs (*v*_*i*_, *v*_*j*_) in which *v*_*i*_ precedes *v*_*j*_ in the total ordering ≺ equal to (*v*_3_, *v*_4_, *v*_1_, *v*_2_). The reference set is *ℛ* = {*ℛ*_3,4_, *ℛ*_3,1_, *ℛ*_3,2_, *ℛ*_4,1_, *ℛ*_4,2_, *ℛ*_1,2_}. In this case a trivial Bayesian network without arrows is equivalent to the prior distribution in (19) and (20) if conditional probability tables are defined by the same values specified in (20) for each feature; that is, features are marginally independent. To see this, note that the context puts a sharp constraint on the space of structures and that the cardinality of the equivalence classes for each configuration *r* of reference features is equal to one; that is,

(21) $$\begin{array}{c} p\left(z\;|\;\xi \right)=\prod_{i=1}^{n_{f}}P\left[R_{i}=r_{i}^{\left[z\right]}\;|\;\xi \right]. \end{array}$$

The formal approach proposed in this paper allows much more flexibility, for example, by restricting the set of nodes to be ordered and by introducing dependence among arrows. For example, let *V*_*B*_ = (*v*_3_, *v*_4_, *v*_1_, *v*_2_) ⊂ *V* be an ordered subset of nodes taken from BC case study. The reference feature *ℛ*_*o*_ = “*V*_*B*_ is the order on the relevant subset of nodes” induces two equivalence classes, the first made by DAGs on *V* which do not satisfy *ℛ*_*o*_, the second one is made by structures without arrows violating the left-to-right order defined in *V*_*B*_. Besides the sharp constraint obtained by setting *P*[*R*_*o*_ = 1 | *ξ*] = 1, the expert might consider such feature uncertain, thus preferring a degree of belief in the set (0,1). Further features could be defined to build the reference set:

(22) $$\begin{array}{c} ℛ={ℛ}_{o}\cup \left\{{ℛ}_{3,4},{ℛ}_{3,1},{ℛ}_{3,2},{ℛ}_{4,1},{ℛ}_{4,2},{ℛ}_{1,2}\right\} \end{array}$$

and the ordered partition

(23) $$\begin{array}{c} 𝒪=\left(\left\{R_{o}\right\},\left\{R_{3,4},\ldots ,R_{1,2}\right\}\right) \end{array}$$

captures weaker causal relationships by features like *ℛ*_*i*,*j*_ = “Variable *X*_*v*_*i*__ is a causal ancestor of *X*_*v*_*j*__.” A CG model made by two components makes possible to elicit conditional beliefs about *v*_4_ → *v*_2_ given the presence of an arrow *v*_3_ → *v*_4_ and given the lack of *v*_3_ → *v*_1_, without assuming a strict order relation on nodes in *V*.

### 3.2. Heckerman et al. (1995)

In Heckerman et al. [9], a prior network *z*_*𝒫*_ was elicited and compared to a candidate network *z* by counting the number of different edges, *δ*, with a high degree of belief assigned to structures closely resembling the prior network. The authors suggested to elicit the hyperparameter 0 < *k* < 1 and to define the prior distribution to be proportional to *k* ^*δ*^.

Among the limitations penalizing the use of this prior we found the following:

- The impossibility of specifying the degree of belief if it depends not only on the number of different edges *δ* but also on their position and type; the presence/absence/direction of an arrow may have an impact on the belief about other edges.
- The elicitation about a subset of *V* is not addressed.
- The causal semantic is natural for this approach because each arrow represents an immediate cause; it seems difficult to mix probabilistic and causal beliefs by counting differences in arrows because a DAG in the probabilistic semantic is just a member of an equivalence class of DAGs representing the same collection of conditional independence relationships.

A simple reformulation is oriented to computation and it involves three operators: arrow deletion, insertion, and change of direction. The reference set of atomic features is *ℛ* = {*ℛ*_*j*_(*z*) : *j* = 0,1, 2,…, *J*}∪{*R*_*a*_(*z*) : *a* = *J* + 1} with *ℛ*_*j*_(*z*) = “The application of *j* operations produces *z*_*𝒫*_,” and where *R*_*a*_(*z*) = “The application of *a* or more operations produces *z*_*𝒫*_.” An undirected graph made by just one clique is associated with potentials represented in Table 3, where *J* = 3. On the right of Table 3, values of the potential function are shown. The normalization constant is *σ* = (*θ*_0_ + *θ*_1_ + *θ*_2_ + *θ*_3_ + *θ*_*a*_)^−1^. It is obviously possible to make the two approaches as close as desired by setting *θ*_*i*_ ∝ *k* ^*i*^ with *i* ≤ 3.

An even simpler reformulation exploits the incompatibility of the above features and it is based on just one reference feature: *ℛ*_1_(*z*, *a*) = “The application of *a* operations produces *z*_*𝒫*_,” with *a* ∈ *𝒜*_1_ = ({0}, {1},…, {4,5,…}), with arrow manipulations (insert-delete-change) defined as above. Feature *ℛ*_1_ essentially defines a plausible neighborhood with respect to a prior network. Further reference features may be introduced to refine such plausibility, for example, by also considering one causal, *ℛ*_2_, and one C.I., *ℛ*_3_, relationships. In other words, a candidate network “close” to the prior network could be associated with a high prior probability which is then tuned according to the presence/absence of two other relevant features. A natural ordered partition on descriptors could be *𝒪* = ({*R*_1_}, {*R*_2_, *R*_3_}); thus a two-component chain graph model may support the elicitation.

A different kind of reformulation is based on the full-probabilistic semantic in which a prior DAG *z*_*𝒫*_ is just a way to define a collection of C.I. relationships. In this case, it is natural to define a structural feature for each conditional independence relationship if it is relevant according to the expert among those represented by *z*_*𝒫*_. The reference set *ℛ* = {*ℛ*_1_,…, *ℛ*_*n*_*f*__} in this case is a collection of plausible C.I. statements taken from the prior DAG. Note that, although it is not essential to draw such prior DAG, it may be useful because a general collection of C.I. statements does not necessarily imply the existence of a compatible DAG.

If none among the C.I. relations is preeminent the ordered partition of descriptors contains just one element, say *𝒪* = ({*R*_1_,…, *R*_*n*_*f*__}), and a CG model made by one component (undirected graphical model) is suited for the elicitation.

### 3.3. Imoto et al. (2003)

In the seminal paper of Imoto et al. [25], the authors developed a framework for combining microarray data and biological knowledge while learning the structure of a BN representing relationships among genes. The proposed criterion has two components, and the second one is particularly interesting because it captures the a priori biological knowledge.

Following their original notation with minor modifications, *π*(*G*) is the prior distribution of network *G*. Then, the interaction energy *U*_*i*,*j*_ of the edge from (gene) *v*_*i*_ to (gene) *v*_*j*_ is defined on a sample space which is categorized into *I* values, say *H*_1_, *H*_2_,…, *H*_*I*_. For example if gene *v*_*i*_ regulates gene *v*_*j*_ then *U*_*i*,*j*_ = *H*_1_ > 0, but if not much is known about such potential regulation then *U*_*i*,*j*_ = *H*_2_ > *H*_1_. The total energy of a network *G* is *E*(*G*) = ∑_(*v*_*i*_,*v*_*j*_)∈*G*_ *U*_*i*,*j*_; thus the sum is taken over existing edges in *G*. The total energy may be rewritten by collecting the parents of each node; thus *E*(*G*) = ∑_*v*_*j*_∈*V*_∑_*v*_*i*_∈*pa*(*v*_*j*_)_ *U*_*i*,*j*_ = ∑_*v*_*j*_∈*V*_ *E*_*j*_. The (prior) probability of network *G* is modeled by the Gibbs distribution:

(24) $$\begin{array}{c} \pi \left(G\right)=\sigma^{-1}\exp\left(-\zeta E\left(G\right)\right), \end{array}$$

where *ζ* > 0 is a hyperparameter and *σ* is the normalizing constant, also called the partition function *σ* = ∑_*G*∈*𝒢*_*V*__exp⁡(−*ζE*(*G*)), with *𝒢*_*V*_ being the collection of all DAGs on a fixed set of nodes *V*.

Operationally, the prior information is coded into a square matrix *U* of size defined by the number of genes, with each *u*_*i*,*j*_ corresponding to *ζH*_1_ or *ζH*_2_ according to the prior belief. Beliefs in protein-protein interactions are coded by *u*_*i*,*j*_ = *u*_*j*,*i*_ = *ζH*_1_. Protein-DNA interactions between the transcription regulator *v*_*i*_ and the controlled gene *v*_*j*_ are accounted by setting *u*_*i*,*j*_ = *ζH*_1_ and *u*_*j*,*i*_ = *ζH*_2_. Some genes are controlled by a transcription regulator through a consensus motif in their DNA promoter region. If genes *v*_*j*_1__, *v*_*j*_2__,…, *v*_*j*_*n*__ have the consensus motif and they are regulated by gene *v*_*i*_ then *u*_*j*_1_,*i*_ = ⋯ = *u*_*j*_*n*_,*i*_ = *ζH*_2_ and *u*_*i*,*j*_1__ = ⋯ = *u*_*i*,*j*_*n*__ = *ζH*_1_.

The seminal approach of Imoto et al. [25] suffers of two main limitations. They stated that the biological knowledge should suggest the partitioning of the underlining continuous energy function but it is not clear how, even after invoking the metaphor of energy from physics. In Example 3.2, they tried *ζH*_1_ = 0.5 (but also *ζH*_1_ = 1) and optimized the selection of *ζH*_2_ = 2.5, a procedure not much in line with pure preexperimental Bayesian elicitation. Moreover, the sum in the partition function is taken on the set of DAGs on *V*, and it becomes quite intractable from 5 nodes on. It follows that their approach is substantially a way to build a score function in a spirit similar to [26], but without providing a flexible support for the calibration of the score function.

The prior information considered by these authors can be also expressed in terms of reference features, for example, by considering features like *ℛ*_*i*,*j*_ = “Gene *v*_*i*_ regulates gene *v*_*j*_,” for all relevant pairs of genes. If preeminent features are absent, an UG model formally resembles the expression based on energy functions but with some major differences:

1. the parameterization is related to subjective probability through odds values;
2. the normalization constant is easier to calculate, at least if the number of features is less than the total pair of genes (some genes omitted);
3. the general calibration constant *ζ* disappears, although a similar tuning has been considered elsewhere [21] to smooth raw elicited odds values while trying to compensate for the well known human tendency towards overstating odds values.

### 3.4. Werhli and Husmeier (2007)

The authors [27], building on the work of Imoto et al. [25], defined a prior information matrix *B* whose elements *B*_*i*,*j*_ ∈ [0,1], with *i*, *j* being a pair of integers for nodes *v*_*i*_ and *v*_*j*_ and the relation *v*_*i*_ → *v*_*j*_. If no prior preference about the presence of such arrow is elicited, then *B*_*i*,*j*_ = 0.5; if 0 ≤ *B*_*i*,*j*_ < 0.5 elicited beliefs put more plausibility on the lack of arrow *v*_*i*_ → *v*_*j*_; if 0.5 < *B*_*i*,*j*_ ≤ 1 higher plausibility favors the presence of the arrow *v*_*i*_ → *v*_*j*_. Note that elements *B*_*i*,*j*_ in *B* are not probabilities.

The calculation of the prior probability for a candidate DAG *G* is straightforward if it is represented through an adjacency matrix in which the element *G*_*i*,*j*_ in row *i* and column *j* is 1 if the DAG has an arrow form *v*_*i*_ to *v*_*j*_, and it is zero otherwise. At first the energy of the DAG is calculated as

(25) $$\begin{array}{c} ℰ\left(G\right)=\sum_{i,j}\left|B_{i,j}-G_{i,j}\right| \end{array}$$

with *G*_*i*,*j*_ and *B*_*i*,*j*_ being, respectively, the elements of *G* and *B*. The probability elicited for *G* is

(26) $$\begin{array}{c} \pi \left(G\;|\;\beta ,B\right)=\sigma^{-1}\left(\beta ,B\right)\exp\left(-\beta ℰ\left(G\right)\right), \end{array}$$

where *β* is a hyperparameter regulating the overall strength of the elicited degree of belief and with *σ* being the partition function which depends on a sum of energy values over the collection of all DAGs on a fixed set of nodes *V*. It follows that an increase of energy is related to a larger mismatch with the prior *B*. In the limit of *β* → 0 a noninformative prior is obtained, while for *β* diverging to infinity peaked priors are defined.

The relationship with the seminal approach of Imoto et al. [25] is clear but despite the easier parameterization chosen to define the energy function, several limitations are still present. First of all, the overall strength *β* is not straightforwardly related to (subjective) probability; therefore at some point the expert has to play numerically with fake examples to get the feeling on reasonable values for such hyperparameter. The calibration of the whole approach is difficult because it depends on the calculation of the normalization constant which is hard to obtain due to the calculation of energy values for all DAGs on a fixed set of nodes *V*. Despite the shortcut proposed by the authors, the calculation of the normalization constant remains as a bottleneck of the approach.

The authors increased the flexibility of their approach in representing prior beliefs by using two matrices *B* ^(1)^ and *B* ^(2)^:

(27) π(G ∣ β1,B(1),β2,B(2))={σ(β1,B(1),β2,B(2))}−1×exp⁡(−{β1ℰ1(G)+β2ℰ2(G)})

with *ℰ*_1_(*G*), *ℰ*_2_(*G*) being the energy functions, respectively, depending on *B* ^(1)^ and *B* ^(2)^. In the elicitation based on a widely adopted database of metabolic pathways, values of *B* are defined as the ratio *m*_*i*,*j*_/*M*_*i*,*j*_, with *M*_*i*,*j*_ being the number of times two genes appear in a pathway and *m*_*i*,*j*_ the number of times that they are linked inside such pathway. Results of a study on 25 genes measured at 73 time points suggested that the procedures using prior information outperformed those without it. Nevertheless, the above mentioned limitations get even worse under such generalization; for example, the explosion in the number of terms entering the partition function must be taken under control by introducing a limitation on the number of arrows entering a node, a technical constraint which may lead to biased elicited beliefs.

The expressivity obtained by these authors through the use of two *B* matrices may be similarly obtained using reference features with some advantages. Back to the BC case study, a major hypothesis under which the elicitation may be performed could be *ℛ*_1_ = “ER is a hub gene,” so that given the configuration *R*_1_ = 1 the expert has to express further conditional beliefs about the other markers; for example, *ℛ*_2_ = “PR regulates P53,” *ℛ*_3_ = “P53 acts on KI67,” *ℛ*_4_ = “P53 acts on NEU.” In this context it is natural to consider the order relation on features *𝒪* = ({*R*_1_}, {*R*_2_, *R*_3_, *R*_4_}); thus a chain graph model is suited for the elicitation with just node *R*_1_ in the first chain component and as many arrows leaving from *R*_1_ as needed to capture changes of conditional beliefs due to a switch in the major hypothesis, from *R*_1_ = 1 to *R*_1_ = 0. Note that, using a chain graph on features, it is possible to tune the flexibility exactly of the amount needed, without the unnecessary and blind consideration of all pairs of genes.

### 3.5. Discussion

The expressivity achieved through reference features is wide whether probabilistic or causal information is elicited. Many limitations found in other approaches depend on the consideration of arrows as the key building block of the elicitation. Further restrictions are due to the use of marginal relationships among arrows, so that severe constraints on the expressivity of the approach follow. The elicitation based on reference features has maximum resolution because, for a suitable set of reference features, it is possible to define a prior distribution characterized by a probability value for each DAG defined on *V*.

There are useful side effects in the approach based on structural features. First, the elicitation effort does not depend on the size of the space of DAGs on *V*, but on the size of the collection of features. Another side effect is related to the cardinality of equivalence classes in *𝒵*. Two candidate DAGs *z*′ and *z*′′ characterized by two different configurations of structural features may receive a very different prior probability value just because the cardinality of the equivalence classes they belong to is very different, say *n*_*r*^[*z*′]^_⋘⋙*n*_*r*^[*z*′′]^_ (see (11)). Note that the cardinality of equivalence classes must be taken into account to preserve probabilistic coherence.

The above description of the elicitation process did not deal with computational issues that are very important for applications. The proposed approach is suited to large networks if the number of DAGs within each equivalence class is available, for example, as a Monte Carlo point estimate. Monte Carlo simulation makes it possible to explore the space of DAGs and it provides evidences about the presence of features which are logically incompatible; thus it may also suggest predicates on which the expert should focus to improve the definition of reference features. Moreover, the elicitation defines a proper prior distribution, so that different MCMC algorithms could be developed to obtain the posterior distribution of *Z*. New greedy search algorithms to find plausible structures in *Ω*_*Z*_ could be investigated to exploit the elicited reference features, for example, by developing an algorithm that generates candidate DAGs belonging to different equivalence classes in *𝒵* at each iteration of the optimization.

The approach to elicitation based on graphical models does not necessitate very esoteric software, although software libraries for platforms commonly adopted in scientific computing would offer the opportunity of performing extensive testing and of investigating human heuristics specifically relevant in this elicitation framework. It is well known that graphical user interfaces facilitate the elicitation, especially if experts are not much trained in Bayesian statistics, and this is a resource which is anyway almost mandatory to face the elicitation in problem domains involving a large number of structural features.

## 4. Conclusions

Graphical models may be exploited to elicit beliefs about the structure of an unknown BN from experts. The joint plausibility on configurations of structural features is decomposed according to conditional independence relationships that are considered plausible by an expert. The expert may use CG models to elicit structural prior information in quite complex domains without leaving a full Bayesian framework. From the elicited CG model, and eventually by using an auxiliary Monte Carlo simulation to estimate the cardinality of equivalence classes, a (proper) subjective prior distribution on the space of DAGs is built and ready to be used with the likelihood function in order to find BN structures supported both by expert beliefs and by collected observations.

No surprise that in complex domains the definition of a prior distribution may be costly. A trade-off should be found by considering the goal of the analysis, how much prior information is available, and the cost and importance of collected data. Here system biology and medicine are expected to be fields in which the proposed approach might be useful, because subjective prior information, besides providing the above mentioned benefits, also tempers the curse of dimensionality caused by structures defined on a high number of variables.

## Figures and Tables

**Table 1:** Elicited vector *a*, with *s* = 5. In the top row, the number *b* of parents is reported, while in the second row the correspondent minimum fraction of nodes *a*_*b*_ is shown.

Number of parents	0	1	2	3	4	5

Cumulative fraction of nodes	0.01	0.2	0.4	0.6	0.8	1.0

**Table 2:** Relation between the original feature (right) and the conjunction of two simpler structural features (left).

Simpler feature 1	Simpler feature 2	Original feature
*ℛ*_1′_	*ℛ*_1′′_	*ℛ*_1_
¬*ℛ*_1′_	*ℛ*_1′′_	¬*ℛ*_1_
*ℛ*_1′_	¬*ℛ*_1′′_	¬*ℛ*_1_
¬*ℛ*_1′_	¬*ℛ*_1′′_	¬*ℛ*_1_

**Table 3:** Potential function for the Heckerman et al. [9] prior.

*R*_0_	*R*_1_	*R*_2_	*R*_3_	*R*_*a*_	Potential
0	0	0	1	0	*θ*_3_
0	0	1	0	0	*θ*_2_
0	1	0	0	0	*θ*_1_
1	0	0	0	0	*θ*_0_
0	0	0	0	1	*θ*_*a*_
Otherwise					0
